# Investigation of Sensitization Potential of the Soybean Allergen Gly m 4 by Using Caco-2/Immune Cells Co-Culture Model

**DOI:** 10.3390/nu13062058

**Published:** 2021-06-16

**Authors:** Ivan V. Bogdanov, Ekaterina I. Finkina, Daria N. Melnikova, Rustam H. Ziganshin, Tatiana V. Ovchinnikova

**Affiliations:** M. M. Shemyakin and Yu. A. Ovchinnikov Institute of Bioorganic Chemistry, Russian Academy of Sciences, Miklukho-Maklaya Str., 16/10, 117997 Moscow, Russia; finkina@mail.ru (E.I.F.); d_n_m_@mail.ru (D.N.M.); ziganshin@mail.ru (R.H.Z.); ovch@ibch.ru (T.V.O.)

**Keywords:** allergen, sensitization, Gly m 4, Caco-2/Immune cells co-culture, cytokine

## Abstract

The soybean allergen Gly m 4 is known to cause severe allergic reactions including anaphylaxis, unlike other Bet v 1 homologues, which induce mainly local allergic reactions. In the present study, we aimed to investigate whether the food Bet v 1 homologue Gly m 4 can be a sensitizer of the immune system. Susceptibility to gastrointestinal digestion was assessed *in vitro*. Transport through intestinal epithelium was estimated using the Caco-2 monolayer. Cytokine response of different immunocompetent cells was evaluated by using Caco-2/Immune cells co-culture model. Absolute levels of 48 cytokines were measured by multiplex xMAP technology. It was shown that Gly m 4 can cross the epithelial barrier with a moderate rate and then induce production of IL-4 by mature dendritic cells *in vitro*. Although Gly m 4 was shown to be susceptible to gastrointestinal enzymes, some of its proteolytic fragments can selectively cross the epithelial barrier and induce production of Th2-polarizing IL-5, IL-10, and IL-13, which may point at the presence of the T-cell epitope among the crossed fragments. Our current data indicate that Gly m 4 can potentially be a sensitizer of the immune system, and intercommunication between immunocompetent and epithelial cells may play a key role in the sensitization process.

## 1. Introduction

Soy-induced allergic symptoms can be systemic and even fatal in some cases [1]. Gly m 4, belonging to the family of Bet v 1 homologues, is one of the most clinically significant allergens isolated from soybeans *Glycine max*, together with other major allergens, such as Gly m 8 [2]. The birch pollen allergen Bet v 1 is a sensitizer responsible for the development of pollen and food allergic cross-reactions. It is known that many other food Bet v 1 homologues tend to cause mild local symptoms, like oral allergy syndrome, in Bet v 1-sensitized individuals [3]. However, Gly m 4 is able to induce severe reactions in allergic patients [4]. That is why Gly m 4 has been selected as a marker allergen for severe food-allergic reactions to soy [5].

Bet v 1 homologues share common structural features including a large internal hydrophobic cavity able to accommodate different ligands *in vitro* [4]. Recently, data supporting a key role of natural ligands binding to allergens in sensitization were reported [6]. Natural ligands of the birch Bet v 1 and hazelnut Cor a 1 allergens–quercetin-3-*O*-sophoroside and quercetin-3-*O*-(2″-*O*-β-d-glucopyranosyl)-β-d-galactopyranoside, respectively, have been identified [7], and an assumption that the natural Bet v 1 ligand can play an important role in the inflammation response has been proposed [8].

The present study aims to elucidate whether the soybean Gly m 4 allergen can be a sensitizer of the immune system. Here, we used quercetin-3,4′-diglucoside (Que-3,4′-di-Glc) as a ligand structurally close to natural ligands of Bet v 1 homologues to evaluate its possible role in a sensitization process. In this investigation, we focused on a possible impact of Que-3,4′-di-Glc on gastrointestinal digestion of Gly m 4 and looked at transport of its fragments through the Caco-2 epithelial barrier and cytokine/chemokine production by immunocompetent cells.

## 2. Materials and Methods

### 2.1. Heterologous Expression of Gly m 4 in E. coli

Recombinant plasmid pET-His8-TrxL-Gly m 4 (6231 bp) was constructed by ligating the 5253 bp BglII/XhoI fragment of pET-31b(+) vector (Novagen) with an insert containing T7 promoter, the ribosome binding site, lac-operator, and the sequence encoding the fusion recombinant protein. The last one included an octahistidine tag, TrxL carrier protein (*E. coli* thioredoxin A with Met37Leu mutation), and mature Gly m 4.0101 sequence [GenBank X60043, UniProt P26987]. The culture of BL21(DE3)/pET-His8-TrxL-Gly m 4 was grown in LB medium with 100 μg/mL ampicillin and 20 mM D(+)glucose at 37 °C. When culture reached OD_600_ of 0.7, expression was induced by the addition of 0.2 mM isopropyl β-D-1-thiogalactopyranoside (Sigma-Aldrich, St. Louis, MO, USA), and incubation was continued for 5 h at 30 °C. The cells, harvested by centrifugation at 6000 *g*, were sonicated on ice in the binding buffer (50 mM Tris-HCl, pH 7.8, 0.5 M NaCl, 20 mM imidazole and 1 mM phenylmethylsulfonyl fluoride (Calbiochem, Los Angeles, CA, USA)). After centrifugation for 20 min at 25,000 *g*, the supernatant containing the soluble fusion protein was collected and loaded onto a Ni^2+^-sepharose (GE Healthcare, Chicago, IL, USA) column, which was prewashed with the binding buffer. The fusion protein was eluted with 0.5 M imidazole and dialyzed overnight against deionized water before lyophilization. Cyanogen bromide cleavage of the fusion protein was performed by using the standard cleavage protocol in 80% trifluoroacetic acid (TFA) (Sigma-Aldrich). In order to purify the target protein from the carrier and unreacted fusion proteins, a repeated IMAC in the same buffer system was performed. Then the target Gly m 4 allergen was purified by two steps of reversed phase high performance liquid chromatography (RP-HPLC). First step was carried out on Reprosil-Pur C18-AQ, d 5 µm, 120Å, 10 × 250 mm (Dr. Maisch GmbH, Ammerbuch, Germany) column by using a linear gradient from 5 to 80% acetonitrile for 60 min with 0.1% TFA at a flow rate of 2 mL/min. Second RP-HPLC step was performed on Luna C18, d 5 µm, 120Å, 4.6 × 250 mm (Phenomenex, Torrance, CA, USA) column by using a linear gradient: 0–40% solution B (0.1% (*v*/*v*) TFA, 80% (*v*/*v*) acetonitrile) for 5 min, 40–60% B for 25 min, 60–100% B for 5 min at a flow rate of 0.7 mL/min.

Endotoxin level was evaluated by the Limulus amebocyte lysate (LAL) test using E-TOXATE Kit (Sigma-Aldrich). The endotoxin level in cell cultures with a final protein concentration was of <0.02 EU/mL.

### 2.2. Ligand-Binding Fluorescence Assay

Gly m 4 was tested for ligand binding by displacement of fluorescent 2-p-toluidinonaphthalene-6-sulphonate (TNS) (Sigma-Aldrich) as previously described [9]. Fluorescence experiments were performed on F-2710 spectrophotometer (Hitachi, Tokyo, Japan). Concentrations of the Gly m 4 and TNS stock solutions were determined spectrophotometrically. A base-line fluorescence of the initial sample of TNS diluted to the concentration of 4 µM with 10 mM phosphate buffer, pH 7.4, was measured by excitation at 320 nm and the emission spectrum was recorded from 330 to 550 nm. Contributions of the buffer, Gly m 4, and the ligand to the measured fluorescence were subtracted. After equilibrating TNS (4 μM) in 10 mM phosphate buffer, pH 7.4, for 2 min with gentle mixing, 2 mM Que-3,4′-di-Glc was titrated into 2 mL of 4 μM Gly m 4 solution in 1 μL aliquots. A simple binding model was employed to express the affinity of the ligand:

F_obs_ = ΔF × (1 − (IC_50/_(IC_50_ + [L])) + F_basiline_, (1)

where F_obs_ is the observed fluorescence, ΔF is the fluorescence change, F_baseline_ is the fluorescence at saturation, and L denotes ligand [10]. IC_50_, ΔF, and F_baseline_ are fitted as free parameters by non-linear least squares regression analysis.

### 2.3. Bioinformatic Approach to Study Interaction of Que-3,4′-di-Glc with Gly m 4

NMR solution structure of Gly m 4 [PDB ID: 2K7H] was used for study *in silico* of the interaction between Gly m 4 and quercetin-3,4′-diglucoside. 3D conformer of Que-3,4′-di-Glc was obtained from the PubChem database [PubChem CID: 5320835]. Preparation of Gly m 4 and Que-3,4′-di-Glc structures for molecular docking was carried out using the DockPrep tool of the UCSF Chimera v.1.4 software package (San Francisco, CA, USA) [11]. The docking box was chosen so that the whole protein molecule in the ribbon representation was entirely inside this box. Blind docking of Que-3,4′-di-Glc based on the Lamarckian genetic algorithm (LGA) into Gly m 4 molecule was carried out using the AutoDock Vina tool of the UCSF Chimera v.1.4 software [12]. The structure of the complex Gly m 4-Que-3,4′-di-Glc was visualized with the Discovery Studio Visualizer v20.1.0.19295 software [13].

### 2.4. Simulation of Gastrointestinal Digestion In Vitro

Gastrointestinal digestion of the recombinant Gly m 4 *in vitro* was performed as previously reported [14]. Briefly, gastric digestion was performed for 2 h using 50 ng (0.1 U) of pepsin (Sigma-Aldrich) per 1 μg of Gly m 4 in 0.05 M HCl, pH 2.0 (final protein concentration 0.05 mM). For duodenal digestion, pH of the mixture resulting from gastric digestion was adjusted to 8.0 by addition of ammonium bicarbonate. The obtained mixture was incubated for 2 h at 37 °C with 2.5 ng (0.03 U) of trypsin (Promega, Madison, WI, USA) and 10 ng (0.4 × 10^−3^ U) of α-chymotrypsin (Sigma-Aldrich) per 1 μg of the substrate. In order to investigate the effect of Que-3,4′-di-Glc on proteolytic cleavage of Gly m 4, the allergen was preincubated with the ligand at protein-to-ligand molar ratio of 1:4 for 10 min. The extent of proteolysis was monitored by sodium dodecyl sulfate polyacrylamide gel electrophoresis [15]. For experiments with cytokines production by cell cultures, 0.5 mg of Gly m 4 was subjected to proteolysis in a similar manner, except duodenal digestion was conducted for 1 h. The obtained digest was frozen at −70 °C. Afterwards, the frozen digest was thawed, diluted with the complete culture medium and used in the experiment.

### 2.5. Human Cell Lines and Cultures

Colorectal adenocarcinoma Caco-2 cell line (ATCC HTB-37) was cultured in complete DMEM/F12 (1:1) medium containing 10% fetal bovine serum (FBS, Invitrogen, Waltham, MA, USA) and 1X antibiotic-antimycotic solution (Invitrogen, Waltham, MA, USA) in a humidified CO_2_-incubator (5% CO_2_, 37 °C). The acute monocytic leukemia THP-1 line (ATCC TIB-202) was cultured in complete RPMI 1640 medium, containing 10% FBS, 1X antibiotic-antimycotic solution, and 0.05 mM β-mercaptoethanol, in the CO_2_-incubator (5% CO_2_, 37 °C).

THP-1 cells were differentiated into pro-inflammatory macrophages (MΦ1) and mature dendritic cells (mDCs) according to previously reported protocols [16,17]. Primary peripheral blood mononuclear cells (PBMC) collected from healthy donor were purchased from American Type Culture Collection (ATCC PCS-800-011), thawed, and seeded into wells of 24- and 96-well plates 2 days prior to the experiment. Two different cell subpopulations (Monocytes and T-/B-/NK-lymphocytes) were isolated from PBMC based on their adherence ability.

For growing cells, mimicking epithelial barriers *in vitro*, Caco-2 cells were seeded onto 24-well polycarbonate Millicell cell culture inserts (0.4 μm, 0.6 cm^2^ surface area) (Millipore, Burlington, MA, USA), precoated with 0.2% bovine gelatin (Sigma-Aldrich), at a density of 7.5 × 10^4^ cells/cm^2^. The cells were grown for 21–29 days in complete DMEM/F12 medium with re-feeding every 2–3 days with a fresh complete medium. The integrity of the Caco-2 cell monolayer was checked by measuring the transepithelial electrical resistance (TEER) using a Millicell-ERS Voltohmmeter (Millipore, Burlington, MA, USA). Only cell monolayers with TEER > 400 Ω cm^2^ (ohm per cm^2^, after subtracting TEER in blank inserts without Caco-2 cells) were used in transport and cytokine production experiments between the 21st and 29th days.

### 2.6. Labeling of Gly m 4 with FITC

The recombinant Gly m 4 was labelled with fluorescein isothiocyanate isomer I (FITC) (Sigma-Aldrich). For this, 1.5 mg of Gly m 4 was reconstituted in 50 µL of DMSO, then added to 300 µL of the buffer for coupling (0.1 M sodium carbonate, 0.1 M sodium bicarbonate, pH 9.6) and 2.9 mg of FITC in 100 µL of DMSO. The coupling reaction was conducted for 2 h at 20 °C in the dark. In order to purify FITC-Gly m 4, the reaction mixture was loaded onto PD10 gel-filtration column (GE Healthcare, Chicago, IL, USA) previously equilibrated with distilled water.

### 2.7. Transport of FITC-Gly m 4 across the Caco-2 Epithelial Barrier

Transport of FITC-Gly m 4 with or without Que-3,4′-di-Glc across the Caco-2 epithelial barrier *in vitro* was performed in the transport buffer (Hank’s balanced salt solution, containing 1 mM MgCl_2_, 1 mM CaCl_2_, and 10 mM D(+)glucose, pH 7.4). Bidirectional “apical-to-basolateral” (A→B) and “basolateral-to-apical” (B→A) transport of FITC-Gly m 4 with or without Que-3,4′-di-Glc in the transport buffer across epithelial barrier was investigated. The “apical-to-basolateral” assay was initiated by adding 0.4 mL of 2 µM Gly m 4 with or without 5 µM Que-3,4′-di-Glc to the apical (luminal) side of the monolayer and 0.7 mL of the transport buffer (pH 7.4) to the basolateral side of the monolayer. The “basolateral-to-apical” assay was performed in a similar manner, except that 0.4 mL of the transport buffer (pH 7.4) was added to the apical side and 0.7 mL of 2 µM Gly m 4 with or without 5 µM Que-3,4′-di-Glc to the basolateral (serosal) side. All solutions were pre-warmed to 37 °C before taking into the transport experiment. Transport *in vitro* across Caco-2 barriers was conducted for 90 min in 4 independent inserts for each studied transport variant (16 inserts in total).

An apparent permeability coefficient (P_app_) was calculated for each insert according to the following equation:

P_app_ = (V/(A × C_i_)) × ΔC/Δt, (2)

where V is a volume of the acceptor chamber, A is the area of the membrane insert, C_i_ is the initial concentration of Gly m 4, ΔC/Δt is the solute flux across the barrier. Uptake ratios:

UR = P_app_(A→B)/P_app_(B→A), (3)

and efflux ratios:

ER = P_app_(B→A)/P_app_(A→B), (4)

for Gly m 4 with or without Que-3,4′-di-Glc were calculated from averaged apparent permeability coefficients measured in 4 independent inserts. Monolayer integrity was checked by measuring TEER before and after the end of the experiment.

### 2.8. LC-MS/MS

Thawed gastroduodenal digest was loaded on a home-made trap column 20 × 0.1 mm packed with Inertsil ODS3 3 μm (GL Sciences, Torrance, CA, USA) in the loading buffer (2% acetonitrile, 97.9% H_2_O, 0.1% trifluoroacetic acid (TFA)) at 10 μL/min flow rate and separated at 20 °C in a home-packed [18] fused-silica column 300 × 0.1 mm, packed with Reprosil Pur C18 AQ 1.9 (Dr. Maisch, Ammerbuch, Germany) and pulled into an emitter using a P2000 Laser Puller (Sutter, Atlanta, GA, USA).

Preparation of each sample from several independent basolateral chambers was performed in the presence of sodium deoxycholate as follows. The sample solution (500 µL) was added to 50 µL of the buffer solution containing 100 mM Tris, pH 8.5, 1% sodium deoxycholate (SDC). The solution was heated at 95 °C for 20 min, cooled to 20 °C, and centrifuged at 16,000 *g* for 15 min. The supernatant was transferred into a preconditioned VIVASPIN spin filter (Sartorius, Göttingen, Germany) with a 10 kDa MWCO PES membrane (cat. no. VS0102). The sample was centrifuged at 15,000 *g* until the volume reached ~50 µL. The filtrate was collected to a clean tube and washed with 200 µL of 0.5 M NaCl. The filter was preconditioned by washing (5 min, 15,000 *g*) with 400 µL of 100 mM Tris, pH 8.5, and then with 400 µL of 100 mM Tris, pH 8.5, containing 1% SDC. The ultrafiltrate was acidified with TFA to the final concentration of 1%. The deoxycholic acid precipitate was extracted with ethyl acetate (3 × 500 µL) under active stirring. Ethyl acetate and the aqueous phase were separated by centrifugation (15,000 *g*, 4 min), upon which ethyl acetate was removed. The peptides contained in the aqueous phase were desalted on Empore SDB-RPS StageTips microcolumns (3M, St. Paul, MN, USA) as described earlier [19], with minor modifications. The samples were applied to a microcolumn (200 *g*, 10 min), and washed with a mixture of 50 µL of 1% TFA and 50 µL of ethyl acetate, then 100 µL of 0.1% TFA. The peptides were eluted with 60 µL of solution containing 5% ammonium hydroxide and 80% acetonitrile. The eluates were spin-dried and stored until the LC-MS analysis at −85 °C.

Reverse-phase chromatography was performed with an Ultimate 3000 Nano LC System (Thermo Fisher Scientific, Waltham, MA, USA), which was coupled to the Q Exactive Plus Orbitrap mass spectrometer (Thermo Fisher Scientific) via a nanoelectrospray source (Thermo Fisher Scientific). The peptides were loaded in a loading solution A (0.1% (*v*/*v*) formic acid, 2% (*v*/*v*) acetonitrile) and eluted with a linear gradient: 3–35% solution B (0.1% (*v*/*v*) formic acid, 80% (*v*/*v*) acetonitrile) for 105 min; 35–55% B for 18 min, 55–99% B for 0.1 min, 99% B during 10 min, 99–2% B for 0.1 min at a flow rate of 500 nl/min. After each gradient cycle, the column was reequilibrated with solution A (0.1% (*v*/*v*) formic acid, 2% (*v*/*v*) acetonitrile) for 10 min. MS1 parameters were as follows: 60 K resolution, 350–2000 scan range, max injection time—30 ms, AGC target—3 × 10^6^. Ions were isolated with 1.4 *m*/*z* window, preferred peptide match and isotope exclusion. Dynamic exclusion was set to 30 s. MS2 fragmentation was carried out in the HCD mode at 17.5 K resolution with the HCD collision energy value of 29%, max injection time–80 ms, AGC target–1 × 10^5^. Other settings: charge exclusion—unassigned, 1, >7.

### 2.9. Cytokines/Chemokines/Growth Factors Production by Cell Cultures

PBMC, T/B/NK, Monocytes, MΦ1 and mDCs were seeded into the wells of 24- and 96-well plates in the complete RPMI 1640 medium 48 h prior to the experiment. Caco-2 cells were seeded into wells of a 96-well plate 3 weeks before the experiment. Then, 24 h after the seeding of all cell lines and cultures, other than Caco-2, into 24- and 96-well plates, Millicell inserts with Caco-2 monolayers with TEER > 400 Ω cm^2^ were placed into the wells of the 24-well plate, containing PBMC, T/B/NK, Monocytes, MΦ1 and mDCs cultures in their basolateral chambers. Then, media in all basolateral chambers were replaced by fresh medium, and each well of the 96-well plate or apical chamber of Caco-2-containing inserts was replaced by fresh complete RPMI 1640 medium with or without compounds under the investigation: fresh medium alone for the control wells, or fresh medium with 5 μM Gly m 4 for 24- and 96-well plates, or fresh medium with 2.5 μM Que-3,4′-di-Glc for the 96-well plate or 5 μM for apical chambers of 24-well plate inserts, or fresh medium with 5 μM Gly m 4 + 2.5 μM Que-3,4′-di-Glc for the 96-well plate or 5 μM Gly m 4 + 5 μM Que-3,4′-di-Glc for apical chambers of 24-well plate inserts, or fresh medium with Gly m 4 digest corresponding to 5 μM of the intact Gly m 4 allergen (Table 1). Cell cultures were kept in CO_2_-incubator (5% CO_2_, 37 °C) for 24 h. Culture supernatants from the 96-well plate and basolateral chambers of 24-well plate were collected 24 h later and stored at −70 °C degrees less than one week prior to analytes assessment. Monolayer integrity was checked by measuring TEER before and after the end of an incubation period.

### 2.10. Assessment of Absolute Levels of Cytokines, Chemokines, and Growth Factors in Cell Cultures

Absolute levels of the following 48 analytes were measured by multiplex xMAP technology using the MILLIPLEX MAP Cytokine/Chemokine/Growth Factor Panel A kit (HCYTA-60K-PXBK48, Merck, Darmstadt, Germany): sCD40L, EGF, Eotaxin-1/CCL11, FGF-2/FGF-basic, Flt-3 ligand, Fractalkine/CX3CL1, G-CSF, GM-CSF, GROα, IFNα2, IFNγ, IL-1α, IL-1β, IL-1RA, IL-2, IL-3, IL-4, IL-5, IL-6, IL-7, IL-8/CXCL8, IL-9, IL-10, IL-12(p40), IL-12(p70), IL-13, IL-15, IL-17A/CTLA8, IL-17E/IL-25, IL-17F, IL-18, IL-22, IL-27, IP-10/CXCL10, MCP-1/CCL2, MCP-3/CCL7, M-CSF, MDC/CCL22, MIG/CXCL9, MIP-1α/CCL3, MIP-1β/CCL4, PDGF-AA, PDGF-AB/BB, RANTES/CCL5, TGFα, TNFα, TNFβ, and VEGF-A. Multiplex-based assay was carried out using MAGPIX system (Merck, Darmstadt, Germany) with the xPONENT 4.2 software (Merck) in accordance with the manufacturer’s instruction. Final analysis was performed with the MILLIPLEX Analyst v5.1 software (Merck). Measurements were performed twice for each sample.

### 2.11. Statistics

Absolute values of the analytes in cell culture supernatants were normalized using a logarithmic transformation by LN function [20] in Microsoft Excel. LN-transformed values were used for comparing the analyte levels in control and experimental samples by unpaired two-sample *t*-test using Statistica v.10.0.1011.0 analytic package (StatSoft, Tulsa, OK, USA). The normality of P_app_ coefficients distribution was assessed using Shapiro-Wilk (*W*-test) and Lilliefors-corrected Kolmogorov-Smirnov tests. P_app_ coefficients for Gly m 4 alone and Gly m 4 with Que-3,4′-di-Glc in both A→B and B→A directions were compared by one-way ANOVA using Statistica v.10.0.1011.0.

## 3. Results

### 3.1. Gly m 4 Is Able to Bind Quercetin-3,4′-Diglucoside

**Figure 1:**
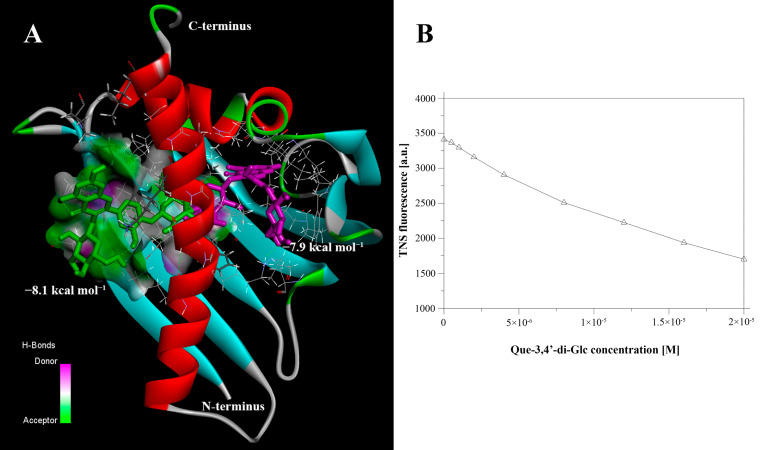
(**A**) Gly m 4 complexed with two best Que-3,4′-di-Glc conformations calculated by means of molecular docking. Green conformation has affinity energy −8.1 kcal mol^−1^, magenta conformation −7.9 kcal mol^−1^. (**B**) Que-3,4′-di-Glc binding to Gly m 4. Titration of 4 µM Gly m 4 and 4 µM TNS with Que-3,4′-di-Glc in 10 mM phosphate buffer, pH 7.4, 25 °C. Fitting the data to Equation (1), IC_50_ yields Kd of 30.2 ± 0.2 μM for Que-3,4′-di-Glc. TNS was excited at 320 nm; the emission at 423 nm for Que-3,4′-di-Glc is displayed.

Previously, it has been shown that Bet v 1 homologues can bind different ligands [21]. To substantiate this finding, we tested Gly m 4 binding with Que-3,4′-di-Glc. At the first stage, the binding of Gly m 4 with Que-3,4′-di-Glc was investigated by means of blind molecular docking. The AutoDock Vina software calculated 10 conformations of the ligand with affinity energy ranges between −8.1 and−6.8 kcal mol^−1^. These two best conformations differed from the others and had lower affinity energies −8.1 and −7.9 kcal mol^−1^, while the rest 8 conformations had affinity energies in the range between −7.3 and −6.8 kcal mol^−1^. In the case of these two most energetically favorable conformations, Que-3,4′-di-Glc is located completely inside the hydrophobic cavity of Gly m 4 (purple) or partially immersed in the cavity near its entrance (green) (Figure 1A). To confirm the ability of Gly m 4 to bind Que-3,4′-di-Glc, we used an extrinsic fluorescent probe, TNS (Figure 1B). TNS is highly fluorescent when bound to the hydrophobic cavity of the protein and competed with lipid molecules for binding with the allergen.

### 3.2. Gly m 4 Can Effectively cross the Caco-2 Epithelial Barrier

**Figure 2:**
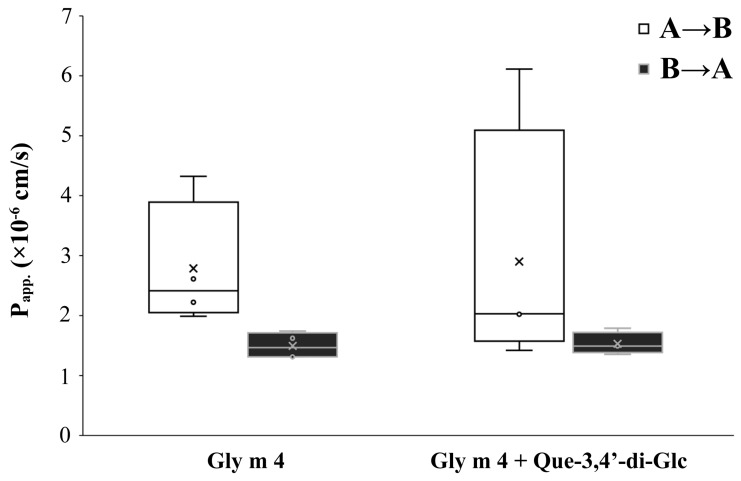
Bidirectional “apical-to-basolateral” (A→B) and “basolateral-to-apical” (B→A) transport of Gly m 4 across the Caco-2 epithelial barrier.

It is known that polarized Caco-2 monolayers represent a reliable model for studies of absorption of drugs and other compounds after oral intake in humans [22]. Proteins labelled with fluorescent probes are widely used for an assessment of permeability of Caco-2 monolayers mimicking the gastrointestinal epithelial barrier [23,24]. Here, we used the FITC-labelled recombinant allergen Gly m 4 for an assessment of “apical-to-basolateral” (A→B, absorptive) and “basolateral-to-apical” (B→A, secretory) bidirectional transport of the allergen across the Caco-2 epithelial barrier. After 90 min around 0.3 μg of Gly m 4 was transported from apical to the basolateral side of the monolayer. Apparent permeability A→B coefficients (P_app_) for Gly m 4 alone measured in 4 independent inserts were within the range of 2–4.5 × 10^−6^ cm/s (Figure 2), which predicts a moderate transepithelial absorption of the Gly m 4 allergen in human gut. The established relationship between the *in vivo* absorption of drugs in humans and P_app_ values allows to correlate P_app_ values ~1–10 × 10^−6^ cm/s with a 20–70% absorption in gut which could be expected in humans, however, in the case of protein allergens it is still to be validated [25].

The uptake ratios were of 1.88±0.022 for Gly m 4 and Gly m 4 with Que-3,4′-di-Glc which suggests active transport, e.g., endocytosis, of the allergen across the Caco-2 epithelial barrier [26]. At the same time, in both cases much lower P_app_ in the B→A direction was observed. The efflux ratios (ER) of 0.532 ± 0.006 for Gly m 4 and Gly m 4 with Que-3,4′-di-Glc argued for not involving active efflux pumps shown to be present in Caco-2 cells, such as P-glycoprotein (ABCB1), ABCG2 or ABCC2, in the Gly m 4 transport across Caco-2 epithelial barrier. The presence of 5 μM Que-3,4′-di-Glc had no significant effect (*p* = 0.13) on the Gly m 4 permeability across the Caco-2 epithelial barrier in both directions (Figure 2). Neither Gly m 4 nor Que-3,4′-di-Glc affected the monolayer integrity which was checked by measuring of TEER following the end of the experiment.

### 3.3. Gly m 4 Is Susceptible to Proteolytic Cleavage Mimicking Gastrointestinal Digestion In Vitro

**Figure 3:**
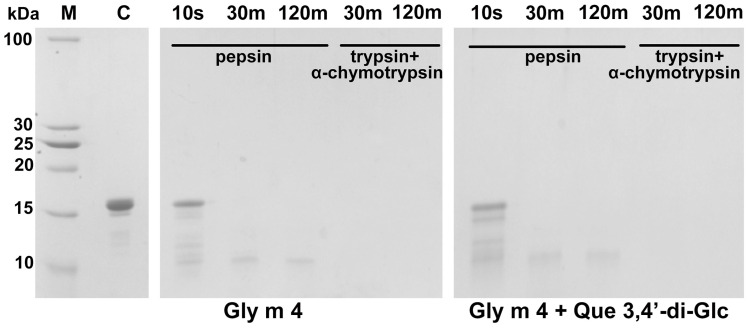
SDS-PAGE analysis of proteolytic cleavage mimicking gastrointestinal digestion *in vitro* of Gly m 4 with or without Que-3,4′-di-Glc. M—molecular mass standards; C—an intact Gly m 4 (control); 10 s, 30 m, 120 m—the allergen fragmentation after incubation with pepsin mimicking gastric digestion during 10 s, 30 min, 120 min, respectively, and digests after the subsequent allergen incubation with the mixture of trypsin and α-chymotrypsin during 30 min and 120 min, respectively.

It is known that Bet v 1 homologues, such as apple Mal d 1, hazelnut Cor a 1, and celery Api g 1 allergens, are rapidly degraded by pepsin during gastric digestion and have moderate susceptibility to trypsin [27]. However, experimental data on the susceptibility of Gly m 4 to gastrointestinal enzymes were not available untill now. Here, Gly m 4 also showed a high susceptibility to cleavage with pepsin mimicking the gastric digestion which resulted in a ~9 kDa fragment that was completely digested by subsequent cleavage with duodenal enzymes *in vitro* (Figure 3). Preincubation of Gly m 4 with Que-3,4′-di-Glc did not affect the rate of gastrointestinal digestion.

**Figure 4:**
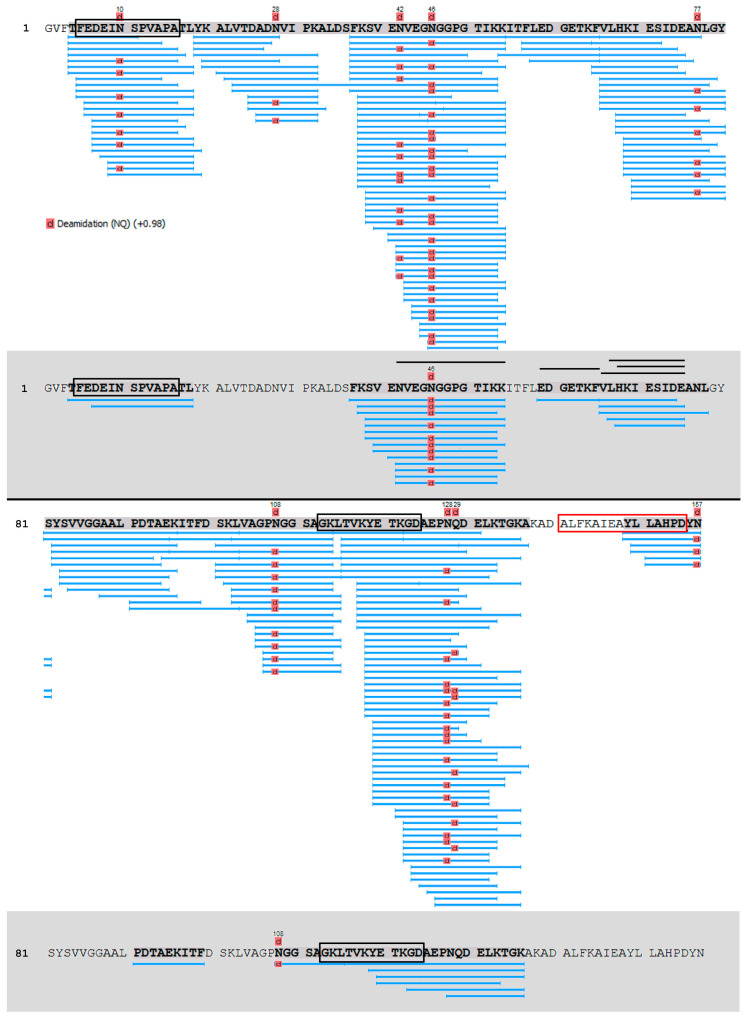
The fragments of Gly m 4 resulted from proteolysis mimicking gastrointestinal digestion *in vitro* (white background), and those ones crossed the Caco-2 epithelial barrier (grey background). Amino acid regions highlighted in bold are denoted sequence, covered by identified LC-MS/MS fragments. Regions, homologous to T-cell epitopes of Bet v 1 able to induce proliferation of Bet v 1-specific T-cell lines from more than 10 patients out of 57 ones, are framed in black, and the immunodominant T-cell epitope of Bet v 1 is framed in red [28]. The most abundant proteolytic fragments of Gly m 4 identified in basolateral chambers are marked with black lines above its sequences.

**Figure 5:**
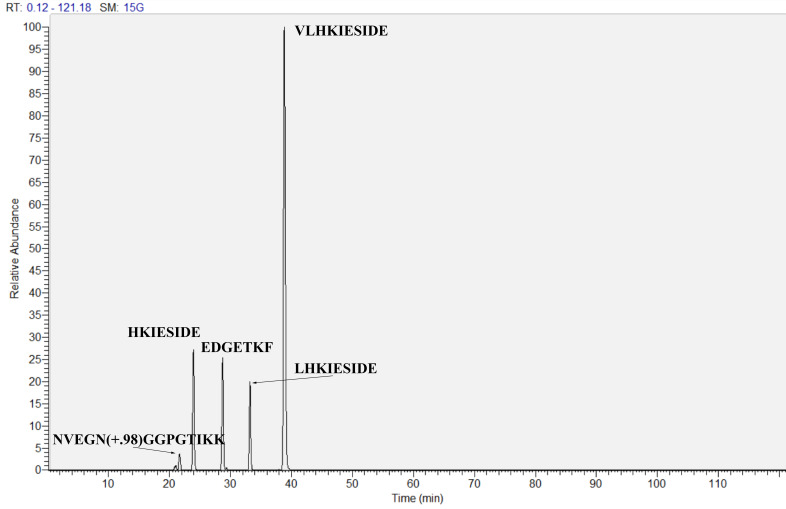
LC-MS/MS chromatogram of the 5 most abundant proteolytic fragments of Gly m 4 identified into basolateral chambers after crossing of the digest through Caco-2 barrier.

We also studied whether resulting proteolytic fragments of the allergen can cross the gastrointestinal epithelial barrier. Gly m 4 proteolytic fragments were analyzed by LC-MS/MS in samples taken from an apical side before and from a basolateral side 24 h after loading the resulted digest onto the insert with the Caco-2 monolayer. Eight clusters of the fragments, covering almost all the amino acid sequence of Gly m 4, have been found after simulated gastroduodenal digestion *in vitro*, which revealed the key sites of the gastrointestinal proteolysis (Figure 4, white background). However, only proteolytic fragments including amino acid residues 4–18, 37–54, 59–77, 91–99, and 104–136 were identified in basolateral chambers after passing of the digest across the Caco-2 monolayer (Figure 4, gray background). T-cell epitopes of birch allergen Bet v 1 have been previously reported by proliferation of short-term allergen-specific T-cell lines (TCLs) derived from a large number of patients (n = 57) with associated food allergy [28]. 7 distinct T cell-activating regions within Bet v 1 were recognized by at least 18% of the studied TCLs [28]. Regions, homologous to two out of these 7 T-cell epitopes, were found among the crossed Gly m 4 proteolytic fragments (Figure 4, in black frames). At the same time, the region, homologous to the immunodominant T-cell epitope Bet v 1_142–156_, which was recognized by 61% of the TCLs, has not been identified in basolateral chambers among the crossed fragments of Gly m 4 (Figure 4, in red frame). Interestingly, the entire region 142–156 homologous to the immunodominant T-cell epitope Bet v 1 was not found after simulated gastroduodenal digestion of Gly m 4 *in vitro*. Among all the identified fragments V_66_LHKIESIDE_75_ had the highest absorptive capacity (Table 2, Figure 5). As Gly m 4 proved to be susceptible to proteolytic enzymes, its digest after cleavage mimicking gastrointestinal digestion *in vitro* was used in the cytokines/chemokines production experiment.

### 3.4. Intercommunication between Epithelial and Immune Cells Changes Cytokine Production in Response to the Intact Gly m 4 and Its Proteolytic Fragments

**Figure 6:**
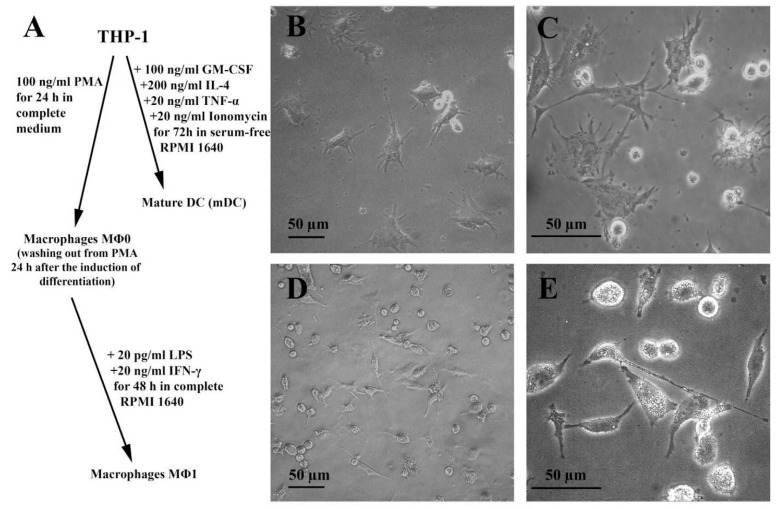
(**A**) Scheme of the differentiation protocol to obtain mature dendritic cells (mDCs) and pro-inflammatory macrophages (MΦ1) from THP-1 cells; Light microscopy of mDCs (**B**,**C**) and MΦ1 (**D**,**E**) cells under magnification of 200× and 400×, respectively.

A pro-monocytic THP-1 line has proved to be a reliable model for obtaining and studying macrophages [16] and mature dendritic cells [17]. Here, we used THP-1 line to differentiate into pro-inflammatory macrophages (MΦ1) and mature dendritic cells (mDCs). Differentiated cells were observed by light microscopy with a CKX41 microscope (Olympus, Tokyo, Japan) equipped with a C310 digital camera and shown to have proper morphological properties (Figure 6). Macrophage MΦ1 polarization was assessed by expression of several classical pro-inflammatory MΦ1 markers, such as cytokines TNFα, IL-1β, IL-6, and chemokine CXCL10 (IP-10) [16]. The analyte levels in MΦ1-containing control well were of 224.9 pg/mL, 119.7 pg/mL, 367.4 pg/mL, and 111.2 pg/mL for TNF-α, IL-1β, IL-6, and CXCL10, respectively, while for THP-1-derived mDCs they were of 37 pg/mL, 14.4 pg/mL, 1.89 pg/mL, and 8.13 pg/mL, respectively (Table S1).

**Figure 7:**
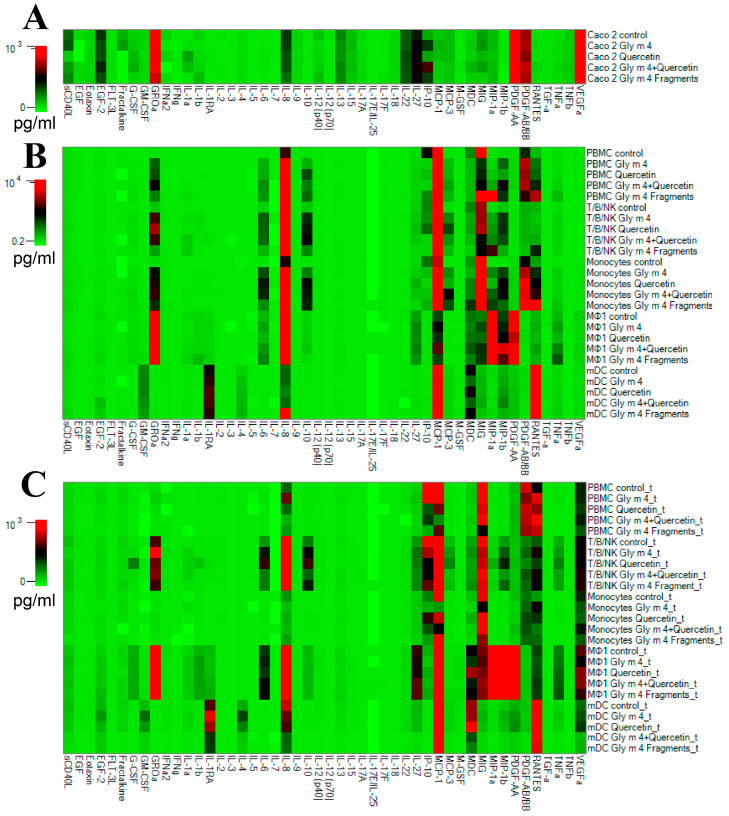
Heat map represented profiles of cytokines/chemokines/growth factors production by (**A**) Caco-2 cell line and (**B**) PBMC, T/B/NK, Monocytes, THP-1-derived MΦ1, and mDCs in response to the direct stimulation, or (**C**) to the transepithelial stimulation by incubation of the same cultures in basolateral chambers of 24-well Millicel inserts after the compounds passed across the Caco-2 epithelial barriers from an apical side.

The experiment on production of the analytes by different cells included two parts. The first one was focused on the study of cytokines, chemokines, and growth factors production by Caco-2 cells (Figure 7A) and various immunocompetent cells (Figure 7B) in response to direct stimulation with Gly m 4, Que-3,4′-di-Glc, the Gly m 4 and Que-3,4′-di-Glc combination, or the Gly m 4 digest resulted from proteolytic cleavage mimicking gastroduodenal digestion *in vitro*. This part of the experiment was carried out into the wells of 96-well plate.

The second part of the experiment consisted of an evaluation of cytokines/chemokines/growth factors production by various immunocompetent cells at the basolateral side of the Caco-2 epithelial barrier after the same studied compounds crossed the barrier from an apical side in a 24-well plate (Figure 7C). Both parts of the experiment were performed at the same time in parallel plates.

**Figure 8:**
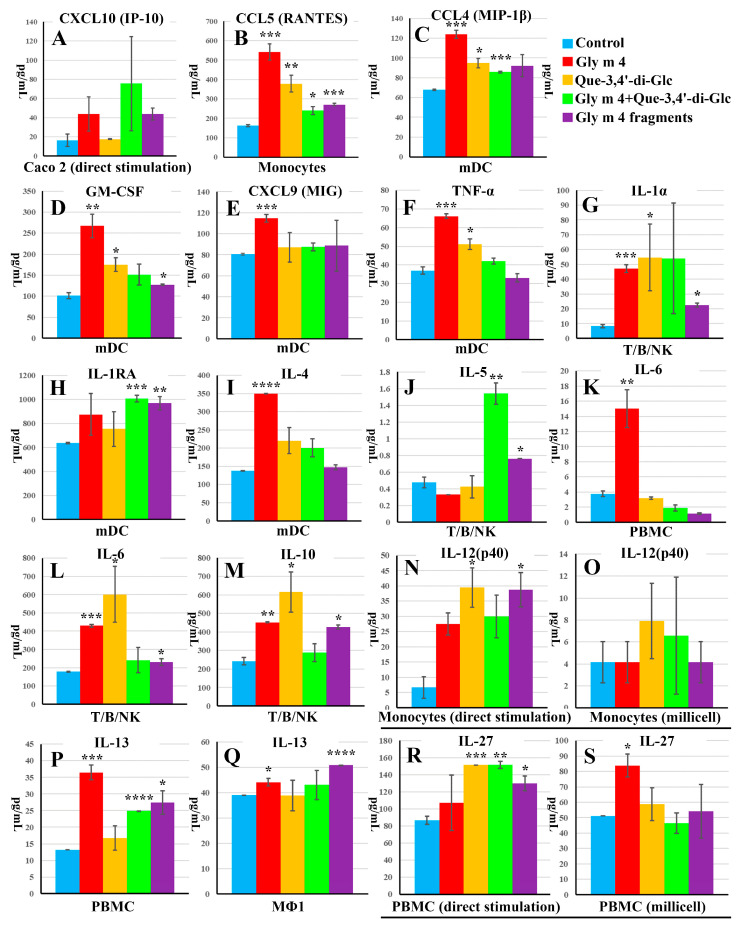
Cytokines and chemokines production by the Caco-2 cell line, Monocytes and PBMC (the direct stimulation) or by PBMC, T/B/NK, Monocytes, MΦ1 and mDCs cultures from basolateral chambers (the transepithelial stimulation). (**A**,**N**,**R**) represent cytokines and chemokines, which were assessed after direct stimulation; (**B**–**M**,**O**–**Q**,**S**) represent cytokines and chemokines, which were assessed after transepithelial stimulation. Error bars represent standard deviation between two technical replications (or biological replications for the direct stimulation). Significance levels are: * *p* < 0.05, ** *p* < 0.01, *** *p* < 0.005, **** *p* < 0.001.

It was shown that both Gly m 4 and its gastroduodenal digest induced production of pro-inflammatory chemokine CXCL10/IP-10 by Caco-2 cells from 16.87 pg/mL in control wells to 44.53 and 43.76 pg/mL in sample wells, respectively (Figure 8A). In Caco-2/immune cells co-culture system Gly m 4 increased production of several pro-inflammatory cytokines and chemokines: RANTES/CCL5 by Monocytes (from 161.32 to 541.41 pg/mL, *p* < 0.005), IL-1α by T/B/NK (from 8.4 to 47.16 pg/mL, *p* < 0.005), IL-6 by PBMC (from 3.76 to 15.02 pg/mL, *p* < 0.01) and T/B/NK (from 130.98 to 769.54 pg/mL, *p* < 0.005), MIP-1β/CCL4 (from 67.89 to 123.8 pg/mL, *p* < 0.005), MIG/CXCL9 (from 80.51 to 114.68 pg/mL, *p* < 0.005), GM-CSF (from 101.52 to 266.73 pg/mL, *p* < 0.01) and TNFα (from 37 to 66.12 pg/mL, *p* < 0.005) by mDCs, as well as anti-inflammatory cytokines: IL-4 by mDCs (from 137.49 to 349.49 pg/mL, *p*<0.001), IL-10 by T/B/NK (from 242.35 to 452.2 pg/mL, *p* < 0.01), and IL-13 by PBMC (from 13.14 to 36.50 pg/mL, *p* < 0.005). Production of the above mentioned pro-inflammatory cytokines and chemokines was not a result of nonspecific activation by residual LPS, which was checked by comparing IL-1β levels in control (12 pg/mL) and Gly m 4-containing (16.61 pg/mL) wells with monocytes in case of direct stimulation, as human monocytes represent a highly pyrogen-sensitive culture. At the same time, in the co-culture system Gly m 4 digest induced increased production of mainly anti-inflammatory cytokines: IL-1 receptor antagonist by mDCs (from 635.14 to 870.41 pg/mL, *p* < 0.01), IL-5 (from 0.48 to 0.76 pg/mL, *p* < 0.05) and IL-10 (from 242.35 to 426.28 pg/mL, *p* < 0.05) by T/B/NK, as well as IL-13 by PBMC (from 13.14 to 27.38 pg/mL, *p* < 0.05) and MΦ1 (from 38.97 to 50.77 pg/mL, *p* < 0.001).

Four patterns of production of cytokines/chemokines/growth factors were observed when comparing the 2 stimulation ways. The first one took place when both direct and transepithelial stimulations did not result in a significant effect on production of analytes. The second one occurred when a level of the same analyte was found to be increased by both stimulation ways compared to control wells. For example, after incubation of PBMC with Gly m 4, the concentration of IL-8 was elevated from 1613 to 7757 pg/mL in case of the direct stimulation and from 290 to 677 pg/mL in case of the transepithelial stimulation. In case of the stimulation with Gly m 4, the same production pattern was observed for IL-10 and IL-1α production by T/B/NK; for IL-6 production by PBMC and T/B/NK; for IL-1α production by Monocytes. The third production pattern was observed when a level of the same analyte was increased by the direct stimulation but remained unchanged when the transepithelial stimulation was carried out. In case of the stimulation with Gly m 4, this pattern was observed for G-CSF production by PBMC; for MCP-3 and IL-1β production by T/B/NK; for IL-6, IL-12(p40) and TNF-α production by Monocytes; for MIP-1α production by PBMC and Monocytes (Figure 8).

The Gly m 4 digest induced the strongest production of TNF-α by PBMC, Monocytes and MΦ1 cultures among all studied compounds by the direct stimulation; however, this effect was not observed in case of the transepithelial stimulation (Table S1). The last production pattern was observed when production of the same analyte remained unchanged after the direct stimulation but was increased in response to the transepithelial stimulation. For instance, in case of the transepithelial stimulation by Gly m 4, this pattern was observed for sCD40L, EGF-2, IL-1α, and IL-1β production by MΦ1; for IL-13 production by PBMC and MΦ1; and for IL-4, G-CSF, and GM-CSF production, by mDCs (Figure 7B,C). These changes in cytokines/chemokines production can be explained by communication between epithelial and immunocompetent cells in the Caco-2/immune cells system by soluble factors. Thus, using the Caco-2/immune cells co-culture model in study of food allergens makes the obtained results more reliable in context of the situation *in vivo*.

## 4. Discussion

The soybean allergen Gly m 4 is known to cause severe allergic reactions including anaphylaxis, unlike other Bet v 1 homologues, which mainly induce local allergic reactions [4]. This work aimed to elucidate mechanisms underlying the unique properties of this allergen. Complexity of the mucosal immune system causes difficulties in mimicking its properties *in vitro*, but a co-culture system makes it possible to elaborate mechanisms involved in communication between epithelial and immune system cells. The co-culture of Caco-2/immune cells was used in current study as a model system [29].

The Gly m 4 allergen can effectively pass across the Caco-2 polarized monolayer which was used in current study as a simplified model of the intestinal epithelium, and then can activate immunocompetent cells. Sensitization effects of Gly m 4 were interpreted according to data obtained by using the Caco-2/Immune cells co-culture as follows. First, passing of the allergen across the Caco-2 barrier activates epithelial cells that resulted in production of pro-inflammatory chemokine CXCL10/IP-10 (Figure 8A), which could activate and recruit leukocytes such as T-cells, eosinophils, monocytes, and NK-cells [29]. CXCL10 was previously proposed to play a role in chronic allergic inflammation [30]. Then, the invaded Gly m 4 might force dendritic cells (DCs), localized underneath the epithelium, to produce CCL4/MIP-1β (Figure 8C), CXCL9/MIG (Figure 8E), which predominantly mediated lymphocytic infiltration to the focal sites, as well as to promote TNF-α production (Figure 8F). These cytokines, apparently, may cause an allergic inflammation in the human gut after Gly m 4 invasion. The increase of CCL4/MIP-1β and CCL5/RANTES obtained in the current research was comparable with their observed increase in biological fluids during allergic inflammation *in vivo* [31,32]. The Gly m 4-induced inflammation might be sustained via IL-1α (Figure 8G), IL-6 (Figure 8K,L) and CCL5/RANTES (Figure 8B), produced by lymphocytes recruited through CXCL10 and CXCL9, and via GM-CSF produced by DCs (Figure 8D). Later on, recruited lymphocytes might inhibit pro-inflammatory stimuli by IL-13 (Figure 8P,Q) [33]. At the same time, Gly m 4-stimulated mDCs produce a key Th2-associated cytokine—IL-4 at a high level (Figure 8I). Being activated by the Gly m 4 allergen, IL-4-producing mDCs apparently move to lymph nodes for the antigen presentation to naïve T-cells with subsequent differentiation of the latter into allergen-specific Th2-lymphocytes. The suggested mechanism coincides with the assumption that the Gly m 4 allergen is potentially able to induce sensitization in a lymph node after absorption in the human gut.

**Figure 9:**
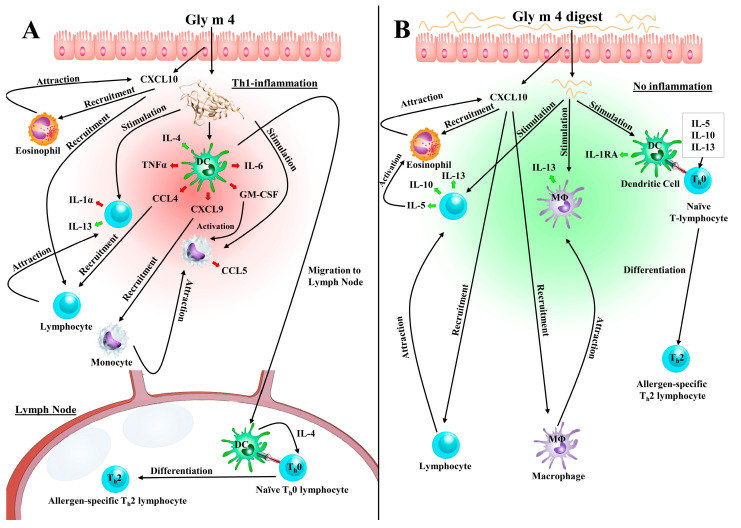
Two possible ways of sensitization with the soybean allergen Gly m 4 based on experimental data obtained in the current study. (**A**) If Gly m 4 can reach the human intestine in intact form, it is able to cross the intestinal epithelium and induce the production of several pro-inflammatory stimuli by different cells, as well as high levels of IL-4 by DCs. Gly m 4-stimulated DCs migrate to the lymph node from intestinal Th1-inflammatory site and induce there IL-4-dependent differentiation of naïve Th0 lymphocytes into allergen-specific Th2 lymphocytes. (**B**) The second proposed mechanism of sensitization is mediated by proteolytic fragments of Gly m 4 that resulted after gastrointestinal digestion. Some of the proteolytic fragments are able to cross the intestinal epithelium and induce the production of several anti-inflammatory stimuli, namely, (IL-1RA), IL-5, IL-10, and IL-13, which leads to the differentiation of naïve Th0 lymphocytes into allergen-specific Th2 lymphocytes in the intestinal lamina propria.

However, in this regard, a key question arises: whether Gly m 4 can reach the intestinal epithelium in its intact immunogenic form? It is known that binding of allergens with ligands may affect their properties and allergenicity. In our study, Que-3,4′-di-Glc had no significant effect on gastrointestinal digestion of Gly m 4, its transport across epithelium and production of cytokines, except IL-5 produced by T/B/NK cells. However, this cytokine by itself can apparently induce only eosinophilic inflammation [34]. Nevertheless, the sensitizing capacity of food allergens may depend, on the one hand, on their susceptibility towards proteolysis in the digestive tract and, on the other hand, on the abundance of T-cell epitopes with immunostimulating capacity [35]. Gly m 4 proved to be susceptible to gastrointestinal enzymes, which provided an evidence that it hardly could reach the intestinal epithelium *in vivo* in an intact form. However, the question is still open. Although some proteolytic fragments resulting from the gastrointestinal digestion of Gly m 4 are capable to pass through the epithelial barrier, they failed to induce IL-4 by mDCs and most of the abovementioned pro-inflammatory stimuli, except CXCL10/IP-10 produced by Caco-2 cells. Instead, proteolytic fragments of Gly m 4 able to cross the Caco-2 monolayer were found to be responsible for a strong anti-inflammatory response by induction of IL-1 receptor antagonist (IL-1RA), IL-5, IL-10, and IL-13 by MΦ1 or lymphocytes (Figure 8H,J,M,P,Q) that can be recruited by CXCL10. These anti-inflammatory stimuli could be responsible for the differentiation of naïve Th0 cells into Th2 after presentation of the crossed fragments by macrophages or dendritic cells in the human gut. Interestingly, Gly m 4 digest induced production of Th2-suppressing cytokines IL-12(p40) and IL-27 by direct stimulation, while transepithelial stimulation did not result in production of these cytokines (Figure 8N,O,R,S) [36,37]. It still remains unclear whether the observed suppression is induced by those Gly m 4 fragments which cannot cross the Caco-2 monolayer or intermediated by epithelial-immune cells communication. Strong anti-inflammatory response of immunocompetent cells toward those Gly m 4 fragments which could pass across the Caco-2 monolayer might speak for the presence of the T-cell epitope among the crossed fragments. Amino acid residues of several crossed fragments correspond to previously mapped T-cell epitopes of the birch Bet v 1 but not its immunodominant epitope Bet v 1_142–156_ [28]. However, Gly m 4 may contain its own T-cell epitopes. Our current data argue for an assumption that the Gly m 4 allergen can potentially act as a sensitizer of the immune system (Figure 9); thus, study of a cohort of Gly m 4-sensitized patients without sensitization to Bet v 1 is of special interest.

To verify our finding, mice models of sensitization by an intact Gly m 4 and its proteolytic fragments through oral administration have to be used in further investigation.

## Figures and Tables

**Table 1:** Two stimulation ways, which were applied to each cell culture, except Caco-2 line (only direct stimulation because Caco-2 cells were on the inserts).

Stimulation Way	Control	Gly m 4 Alone	Que-3,4′-di-Glc Alone	Gly m 4 + Que-3,4′-di-Glc	Gly m 4 Digest
Direct stimulation (into 96-well plate)	−	5 μM	2.5 μM	5 μM + 2.5 μM	5 μM
Transepithelial stimulation (into 24-well plate with Caco-2 inserts)	−	5 μM	5 μM	5 μM + 5 μM	5 μM

**Table 2:** Gly m 4 proteolytic fragments identified by LC-MS/MS in basolateral chambers after passing them across the Caco-2 monolayer. The table is ranked based on highest-to-lowest peak area from the top to the bottom.

Peptide	−10lgP	Molecular Mass	ppm	*m/z*	RT	Peak Area
V_66_LHKIESIDE_75_	33.48	1181.6292	0.9	591.8224	38.81	1.50 × 10^8^
H_68_KIESIDE_75_	22.38	969.4767	0.6	485.7459	23.92	2.07 × 10^7^
L_67_HKIESIDE_75_	27.23	1082.5608	1.6	542.2885	33.2	1.76 × 10^7^
E_59_DGETKF_65_	23.74	824.3552	2.1	413.1857	28.72	1.51 × 10^7^
N_42_VEGN(+0.98)GGPGTIKK_54_	33.21	1270.6517	1.9	636.3344	21.63	1.05 × 10^7^
Y_119_ETKGDAEPNQDELKTGK_136_	48.5	2021.9541	3.5	1011.988	27.7	9.75 × 10^6^
K_38_SVENVEGN(+0.98)GGPGTIKK_54_	45.73	1713.8896	2.6	857.9543	25.6	9.26 × 10^6^
S_39_VENVEGN(+0.98)GGPGTIKK_54_	34.66	1585.7947	2.2	529.6067	30.94	8.78 × 10^6^
N_42_VEGN(+0.98)GGPGTIK_53_	27.83	1142.5568	2.5	572.2871	28.81	3.99 × 10^6^
T_116_VKYETKGDAEPNQDELKTGK_136_	35.94	2350.1653	4	784.3989	29.17	2.32 × 10^6^
S_39_VENVEGNGGPGTIKK_54_	31.09	1584.8107	3.1	793.415	29.33	2.30 × 10^6^
F_37_KSVENVEGN(+0.98)GGPGTIKK_54_	39.96	1860.9581	−0.7	466.2465	38.79	2.07 × 10^6^
N_108_(+0.98)GGSAGKL_115_	22.71	703.35	1.2	352.6827	23.07	1.86 × 10^6^
N_42_VEGNGGPGTIKK_54_	26.72	1269.6677	1	424.2303	18.9	1.47 × 10^6^
K_38_SVENVEGN(+0.98)GGPGTIK_53_	41.56	1585.7947	3.3	793.9072	32.29	1.31 × 10^6^
N_42_VEGNGGPGTIK_53_	24.76	1141.5728	1.8	571.7947	26.04	1.01 × 10^6^
V_40_ENVEGN(+0.98)GGPGTIK_53_	22.11	1370.6677	3.3	686.3434	35.55	9.07 × 10^5^
E_120_TKGDAEPNQDELKTGK_136_	46.16	1858.8907	1.4	930.4539	22.67	8.76 × 10^5^
E_41_NVEGN(+0.98)GGPGTIK_53_	30.24	1271.5994	1.8	636.8081	30.92	8.76 × 10^5^
V_66_LHKIESID_74_	20.04	1052.5865	1.6	527.3014	36.84	8.58 × 10^5^
N_128_QDELKTGK_136_	22.69	1031.5247	3.4	516.7714	15.08	7.29 × 10^5^
V_40_ENVEGN(+0.98)GGPGTIKK_54_	24.92	1498.7627	3.2	750.391	27.38	7.26 × 10^5^
T_4_FEDEINSPVAPATL_18_	36.32	1602.7777	3.6	802.399	97.49	5.50 × 10^5^
G_123_DAEPNQDELKTGK_136_	35.57	1500.7056	1.7	751.3613	25.69	5.18 × 10^5^
S_39_VENVEGNGGPGTIK_53_	23.14	1456.7157	5.1	729.3688	38.54	5.00 × 10^5^
P_91_DTAEKITF_99_	30.33	1020.5128	2.5	511.265	64.51	3.78 × 10^5^
S_39_VENVEGN(+0.98)GGPGTIK_53_	27.36	1457.6997	1.6	729.8583	40.55	3.75 × 10^5^
V_66_LHKIESIDEANL_78_	33.81	1479.7932	0.9	740.9045	64.96	2.07 × 10^5^
E_120_TKGDAEPNQDELK_133_	34.3	1572.7267	1.7	787.3719	24.41	1.43 × 10^5^

Peptide—amino acid sequences of the peptides determined by the PEAKS search workflow. A modified residue is followed by a pair of parentheses enclosing the modification mass. −10lgP—the peptide −10lgP score. Ppm—the precursor mass error, calculated as 10^6^ × (precursor mass − peptide mass)/peptide mass. RT—retention time (elution time).

## Data Availability

All data generated and analyzed during this study are included in this published article and its supplementary information file “Table S1”.

## Supplementary Materials

The following are available online at https://www.mdpi.com/article/10.3390/nu13062058/s1, Table S1: Absolute levels of 48 cytokines, chemokines and growth factors assessed by multiplex xMAP technology.
